# Research on the Fractal Characteristics and Energy Dissipation of Basalt Fiber Reinforced Concrete after Exposure to Elevated Temperatures under Impact Loading

**DOI:** 10.3390/ma13081902

**Published:** 2020-04-17

**Authors:** Wenbiao Liang, Junhai Zhao, Yan Li, Yue Zhai

**Affiliations:** 1 Department of Civil engineering, School of Civil Engineering, Chang’an University, Xi’an 710061, China; hqlwb2007@126.com; 2 Department of safety engineering, School of Geology Engineering and Geomatics, Chang’an University, Xi’an 710054, China; zy@chd.edu.cn

**Keywords:** basalt fiber, concrete, elevated temperatures, SHPB, impact velocity, fractal characteristics, energy dissipation

## Abstract

The fractal characteristics and energy dissipation of basalt fiber reinforced concrete (BFRC) with five kinds of fiber volume contents (0.0%, 0.1%, 0.2%, 0.3%, 0.4%) after exposure to different temperatures (20 °C, 200 °C, 400 °C, 600 °C, 800 °C) under impact loading were investigated by using a 50 mm diameter split Hopkinson pressure bar (SHPB) apparatus. Scale-mass distribution rules and fractal dimension characteristics of fragments were studied based on the screening statistical method and the fractal theory. Furthermore, the relationship between the energy consumption density and the fractal dimension of fragments was established, and the effects of fiber content, temperature and impact velocity on fractal dimension and absorption energy were analyzed. The results show that the crushing severity of fragments and fractal dimension increase with the impact velocity under the same fiber content. The energy consumption density increases first and then decreases with increasing fiber content, and also decreases with increasing temperature. When the temperature and fiber content remain unchanged, the energy consumption density increases linearly with the increasing fractal dimension, and under the same impact velocity and temperature, there is no obvious linear relationship between energy consumption density and fractal dimension.

## 1. Introduction

Basalt fibers (BF) are produced from basalt rocks in the process of rock melting, gravitational leakage of molten basalt mass through holes in platinum boats and cooling. The excellent properties of basalt fibers such as outstanding resistance to elevated temperature, superior mechanical properties, good compatibility with cement and concrete and cost performance make basalt fibers a good reinforcing material in concrete composites [1,2,3]. Compared with plain concrete, basalt fiber reinforced concrete (BFRC) has the advantages of crack and impact resistance due to the combination of basalt fibers and concrete, so the engineering application range of BFRC has become more extensive in recent years. Structures in special fields such as underground and protective structures are facing the threat of extreme loads such as impact, explosion and elevated temperature, for example, military protection engineering should consider the effects of high-speed impact loads and high temperatures derived from explosions; the collapse of the building’s upper parts caused by the explosion of chemicals will hit the lower structures at a high temperature. It has been generally accepted that concrete deteriorates and exhibits poor performance after being exposed to elevated temperatures. The coupling effect between impact load and elevated temperature should be considered in the structural design and damage effect analysis, in order to prevent potential safety risks caused by overestimating the bearing capacity of materials. So the research on fractal characteristics and energy dissipation of BFRC after exposure to elevated temperatures under impact loading has an important theoretical value and practical significance.

There have been many studies on the static and dynamic properties of BFRC. These studies are mainly focused on mechanical properties, constitutive models, microscopic composition and other aspects [4,5,6,7,8,9,10,11,12,13], but there are few studies on energy analysis. However, the failure process of concrete is actually an irreversible energy consumption process of energy exchange with the outside, so research on failure behavior of concrete through energy analysis is necessary. Some studies show that the process of concrete impact crushing is a fractal evolution process driven by external energy, and the distribution of the fragments size satisfies a certain fractal characteristics [14,15,16]. Therefore, fractal theory [17] can be used to calculate and analyze the fractal dimension of concrete after impact crushing and study its internal law. Li [14] and Shi [15] et al. conducted split Hopkinson pressure bar (SHPB) tests on concrete under freeze-thaw cycle conditions and exposure to elevated temperature, respectively, and analyzed the fractal dimension, energy dissipation of fragments and the effect of factors. Ren studied the dynamic characteristics and fractal characteristics of BFRC after exposure to elevated temperature, and analyzed the factors affecting dynamic strength and fractal characteristics, such as strain rate, temperature, fiber content and porosity [16]. However, the above research did not study the relationship between the energy dissipation and the fractal characteristics of BFRC after exposure to elevated temperatures under impact loading.

In this paper, the impact test of BFRC with five kinds of fiber volume content (0.0%, 0.1%, 0.2%, 0.3%, and 0.4%) after exposure to different temperatures (20 °C, 200°C, 400 °C, 600 °C and 800°C was conducted using a 50-mm-diameter SHPB apparatus. The fragments for various working conditions were sieved and the scale-mass distribution curves were obtained. Based on the fractal theory and energy dissipation principles of SHPB test, the influences of fiber content, temperature and impact velocity on fractal dimension were analyzed, and the relationship between energy dissipation density and fractal dimension of BFRC was established.

## 2. Experimental Procedure

### 2.1. Materials and Specimen Preparation

The mixing proportions of concrete specimens in this test are shown in Table 1. Ordinary Portland cement with a strength grade of 42.5 was used. The coarse aggregate was 5–16mm continuously graded gravel. The fine aggregate was continuously graded medium size sand with a fineness modulus of 2.42. Basalt fibers were produced by Shanghai Chenqi Chemical Technology Co., Ltd., and their performance parameters are shown in Table 2. The basalt fibers were added into the matrix according to the proportion of volume content of 0.1%, 0.2%, 0.3% and 0.4%. The characteristics of fly ash are as follows: fineness 43μm, density 2.4 g/cm^3^, moisture content about 5%. The pH value of the water reducer ranges from 7 to 9, and the water reduction rate is about 20%–35%. The test water was tap water.

The basalt fiber reinforced concrete was cast in the shape of a 150 mm × 150 mm × 150 mm cube first, and then 50 mm × 25 mm cylinder specimens were made from cubes through a coring and cutting process. The two end faces of the specimen were polished to ensure that their non-parallelism and non-perpendicularity were less than 0.02 mm.

### 2.2. Heating and Cooling

**Figure 1:**
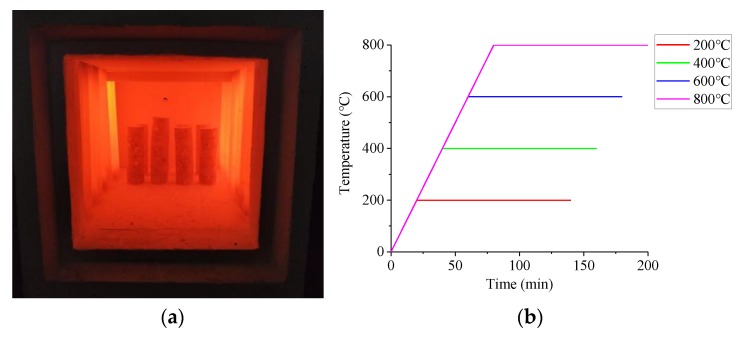
The heating process and heating curve. (**a**) The box-type resistance furnace; (**b**) The heating curve.

The heating equipment was the artificial intelligence box-type resistance furnace produced by Luoyang Sigma Furnace Industry Co., Ltd., Luoyang, China. The temperatures were controlled by the computer, so the process of heating could be automatic and accurate. The target temperatures were 20 °C (room temperature), 200 °C, 400 °C, 600 °C, 800 °C. The heating rate of the resistance furnace was set at 10 °C/min to better simulate the real fire environment. The target temperatures were held constant for 2 h to ensure that the internal and external temperature of specimens become consistent and the chemical and physical changes of specimens were more thorough. The heating process and heating curve of the specimen are shown in Figure 1.

**Figure 2:**
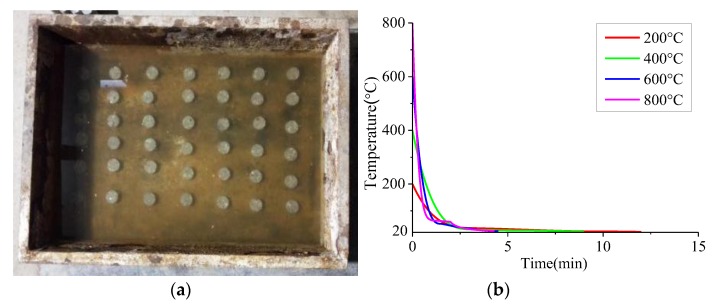
The cooling process and cooling curve. (**a**) The water tank; (**b**) The cooling curve.

In a real situation, a fire is usually extinguished using water, so water cooling was chosen in this study [18]. The specimens were taken out immediately after the heating procedure and put into a 1.0 m × 0.8 m × 0.4 m water tank. The water depth in the water tank was set at 0.3 m to ensure the same cooling water consumption for every specimen and a thermometer was used to measure the change of temperature. The cooling process and cooling curve of the specimens are shown in Figure 2. The specimens after soaking were left in the air for 4 weeks in order to eliminate the influence of water content.

### 2.3. SHPB Tests and Sieve Tests

**Figure 3:**
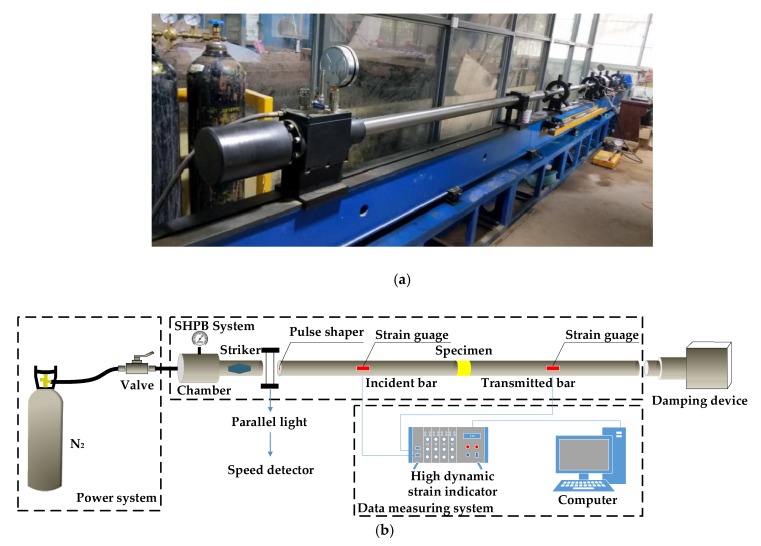
50mm split Hopkinson pressure bar (SHPB) apparatus. (**a**) SHPB photo; (**b**) Schematic diagram.

The dynamic compressive tests of BFRC specimens were conducted by a 50 mm diameter split Hopkinson pressure bar (SHPB, produced by Luoyang Liwei Technology Co., Ltd., Luoyang, China) apparatus, as shown in Figure 3. The length of the incident bar is 2500 mm and the transmitted bar is 2000 mm. The pulse shaping technique [19] was adopted during tests to eliminate the high frequency oscillation, reduce the wave dispersion effects, and to extend the rising time of the incident pulse to ensure that the specimens have enough time to reach the state of stress equilibrium. In this research, a copper sheet was chosen as a pulse shaper. Through the comparative analysis of the tests, the dimensions of the copper sheets were as follows: the thickness was 1mm and the diameters were 25 mm, 20 mm, 15 mm, and the corresponding impact velocities were 5.4 m/s, 8.8 m/s, 11.3 m/s.

**Figure 4:**
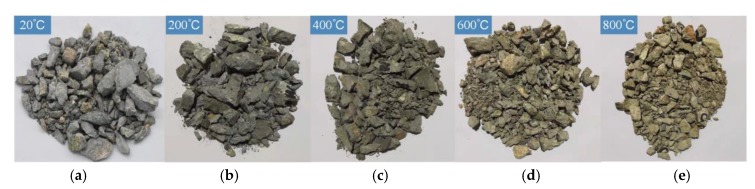
The impact fracture patterns of basal fiber reinforced concrete (BFRC) specimens after exposure to different temperatures. (**a**) 20 °C, (**b**) 200 °C, (**c**) 400 °C, (**d**) 600 °C, (**e**) 800 °C.

Figure 4 shows the impact fracture patterns of BFRC specimens after exposure to different temperatures (the fiber content is 0.2%).

The ZBSX-92A shock type standard vibrating sieve (Xingye Test Instrument Co., Ltd., Cangzhou, China.) was used to screen the concrete fragments collected after impact tests. The mesh diameter of the sieve is 16 mm, 13.2 mm, 9.5 mm, 4.75 mm, 2.36 mm, 1 mm and 0.5 mm, respectively. The sieve swings 221 times and vibrates 147 times per minute, with a swing scope of 25 mm to ensure that fragments of each particle size could be separated effectively through the sieve. Then, the mass of the material under each stage of the sieve was measured by a high-sensitivity electronic scale, and the test data were recorded.

## 3. Fractal Characteristics of Fragments

### 3.1. Scale-Mass Distribution of Fragments

**Figure 5:**
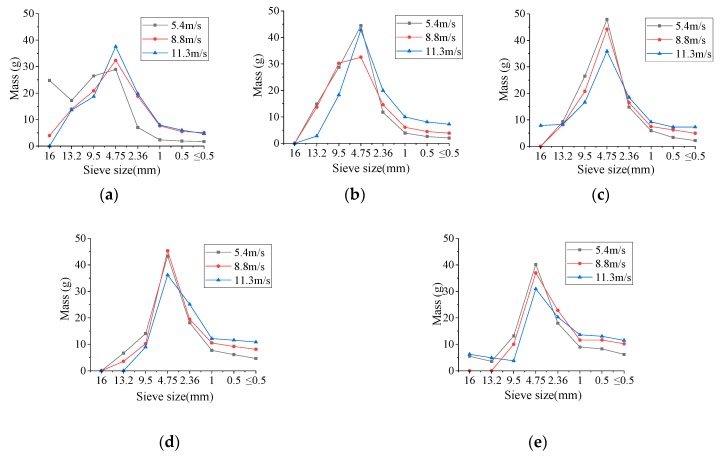
The scale-mass distribution curves for BFRC fragments for various temperatures and impact velocities (the fiber content is 0.2%). (**a**) 20 °C; (**b**) 200 °C; (**c**) 400 °C; (**d**) 600 °C; (**e**) 800 °C.

**Figure 6:**
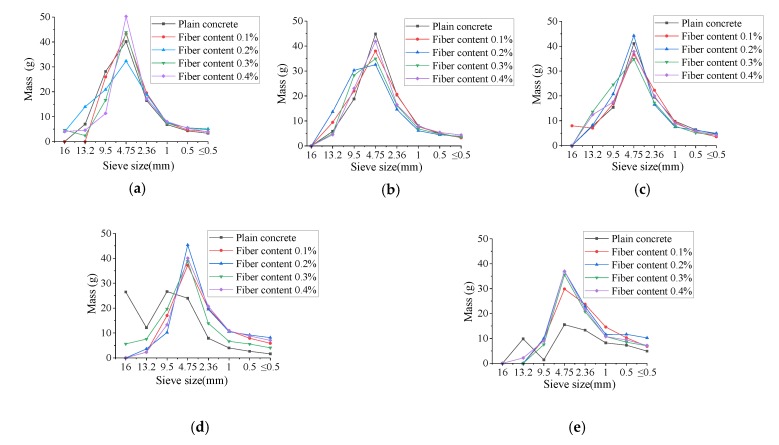
The scale-mass distribution curves for basalt fiber concretes for various temperatures and fiber contents (the impact velocity is 8.8 m/s). (**a**) 20 °C; (**b**) 200 °C; (**c**) 400 °C; (**d**) 600 °C; (**e**) 800 °C.

The scale-mass distribution curves for BFRC fragments for various temperatures and impact velocities are shown in Figure 5 (the fiber content is 0.2%). The scale-mass distribution curves for basalt fiber concretes for various temperatures and fiber contents are shown in Figure 6 (the impact velocity is 8.8 m/s).

It can be seen from Figure 5 that when the fiber content is 0.2%, the scale-mass distribution curve of fragments is approximately a bell curve for different temperatures and impact velocities, and the curve is high in the middle and low at both ends. Specifically, the mass percentages of fragments for the particle size of 4.75–9.5 mm are the highest. For the particles larger than 13.2 mm and smaller than 1mm under every condition the mass percentages are lower.

Figure 5 also indicates that when the fiber content is 0.2%, the peak points of the bell curve gradually decrease with the increase in the impact velocities, and the ratio of large and small particle sizes at both ends gradually increases. For example, when the elevated temperature is 400 °C the impact velocities are 5.4 m/s, 8.8 m/s, and 11.3 m/s, the corresponding mass percentages of fragments for particle sizes of 4.75–9.5 mm account for 43.6%, 40.8%, and 32.4%, respectively; for particle sizes greater than 13.2 mm are 8.5%, 7.6%, and 7.6%, respectively, and for particle sizes less than 1mm are 10.4%, 17.2%, and 21.4%, respectively. Therefore, the larger the impact velocity, the more homogenized the fragment and the more serious the crushing condition.

By comparing Figure 5a–e, it can be seen that the scale-mass distribution of BFRC fragments is similar for various different temperatures, which is still approximate to a bell curve, but the mass percentage varies with different particle size. The scale-mass curve is “dumpy” when the temperature is 20 °C, indicating that the mass distribution of fragments is less discrete and the proportion of the larger fragments is higher. The scale-mass curves are obviously “high and thin” at 200 °C–600 °C, indicating that the discreteness of mass distribution increases, the mass distribution proportion of fragments with different particle sizes varies greatly, and the proportion of fragments with medium particle sizes is the highest. When the temperature reaches 800 °C, the scale-mass curve tends to be "dumpy" again, the mass distribution of the fragments with various particle sizes tends to be uniform again, and the proportion of the fragments with small particle sizes increases significantly. The reason for this phenomenon is that the higher the heating temperature, the greater the damage of BFRC, and the more thorough the crushing.

As can be found in Figure 6, there is no obvious difference of scale-mass curve between plain concrete and BFRC when the temperature is less than 400 °C, when the temperature rises to 600 and 800 °C, the scale-mass curve of BFRC still show the shape of the bell due to the basalt fiber toughening effect and good resistance to high temperature performance. However, for plain concrete, bell curve distribution was no longer obvious due to excessive temperature damage, especially at 800 °C, the quantity of fragments with each size are more homogenization.

### 3.2. Fractal Characteristics of Fragments

According to the mass-frequency relationship [20], the distribution Equation of concrete fragments under the impact loading is

Y = *M_r_/M_T_* = *(r/r_m_)^3 − D^* (1)

where *r, r_m_* and *D* are the particle size, the maximum size and the fractal dimension of crushing blocksrespectively; *Mr* is the total mass of crushing blocks with particle size smaller than *r*; *M_T_* is the total mass of specimen crushing blocks.

Take the natural logarithm on the both sides of Equation (2) to get

lnY = ln(*Mr*/*M_T_*) = (3 − *D*) ln(*r*/*r_m_*) (2)

In the ln (*Mr*/*M_T_*)-ln (*r*/*r_m_*) coordinate system, the slope of the fitting line is *K = 3 − D*. Therefore, the fractal dimension of crushing blocks can be calculated by the mass-granularity method to be

*D* = *3 − K* (3)

**Figure 7:**
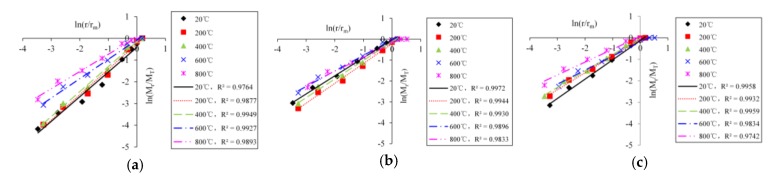
The curves of ln *(M_r_/M_T_)*-ln *(r*/*r_m_)* (the fiber content is 0.2%). (**a**) 5.4 m/s; (**b**) 8.8 m/s; (**c**) 11.3 m/s.

**Figure 8:**
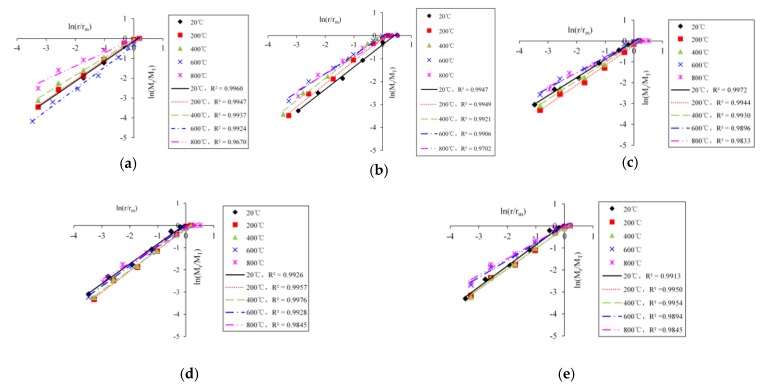
The curves of ln *(M_r_****/****M_T_)*-ln *(r*/*r_m_)* with different temperature and fiber contents (the impact velocity is 8.8 m/s). (**a**) 0.0%; (**b**) 0.1%; (**c**) 0.2%; (**d**) 0.3%; (**e**) 0.4%.

The logarithmic curves of concrete fragments with different temperatures and impact velocities are shown in Figure 7 (the fiber content is 0.2%). The logarithmic curves of concrete fragments with different temperature and fiber contents are shown in Figure 8 (the impact velocity is 8.8 m/s).

As can be seen from Figure 7 and Figure 8, there is a strong linear correlation between ln (*M_r_/M_T_*) and ln (*r*/*_m_*), indicating that the distribution of BFRC fragments has a fractal feature. The reason that BFRC has a fractal feature is that the failure of BFRC is the result of the continuous development of internal mesoscopic cracks and pore under temperature damage and impact load, and the internal mesoscopic cracks and pore distribution conforms to the fractal theory, so fragments of BFRC also show some self-similarity feature and conform to the fractal rule in statistical sense. We could draw a conclusion that the destructive process of BFRC is from microscopic damage to the macroscopic crushing caused by external force. The process is characterized by fractal properties, and the larger the fractal dimension, the greater the number of fragments, the smaller the size, and the more serious the material crushing.

### 3.3. Influencing Factors Analysis of Fractal Dimension

**Figure 9:**
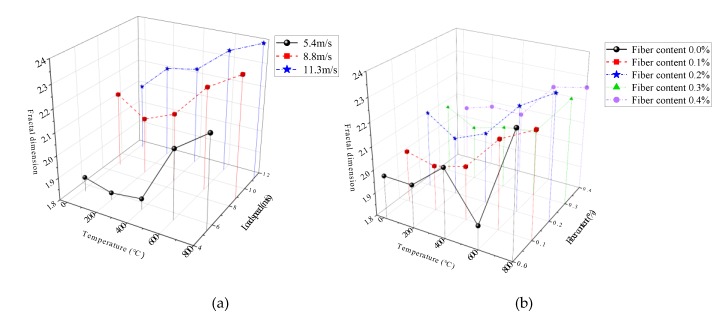
The fractal dimension versus influencing factors. (**a**) temperatures and impact velocities (the fiber content is 0.2%). (**b**) temperatures and fiber contents (the impact velocity is 8.8 m/s).

The relationship of fractal dimension with temperatures and impact velocities is shown in Figure 9a (the fiber content is 0.2%). The relationship of fractal dimension with temperatures and fiber contents is shown in Figure 9b (the impact velocity is 8.8 m/s).

Figure 9a shows that when the fiber content is 0.2%, the fractal dimension of BFRC are 1.87–2.21, 2.13–2.34, and 2.10–2.40, the corresponding impact velocities are 5.4 m/s, 8.8 m/s and 11.3 m/s, respectively, in the temperatures range of 20 °C–800 °C. Within the impact velocities range of 5.4 m/s–11.3 m/s, the fractal dimension of specimens at 20 °C–800 °C is 1.87–2.10, 1.83–2.21, 1.85–2.23, 2.11–2.34, 2.21–2.40, respectively. It can be seen that the fractal dimension increases significantly with the increase in impact velocities and temperature, namely, the higher the temperature, or the higher the loading rate, the higher the fracture degree of specimens.

**Figure 10:**
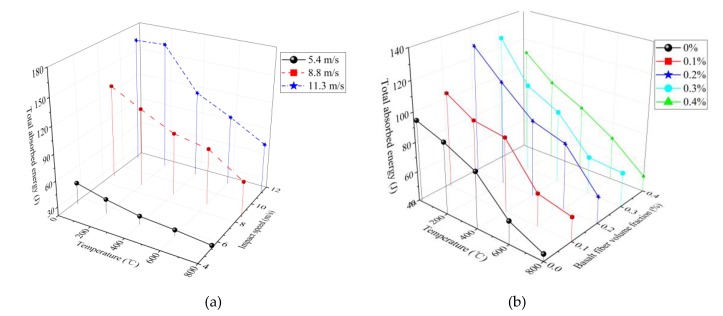
Absorbed energy versus influencing factors. (**a**) temperatures and impact velocities (the fiber content is 0.2%); (**b**) temperatures and fiber contents (the impact velocity is 8.8 m/s).

Figure 9b shows that at the same impact velocity, the fractal dimension from 20 °C to 200 °C changes little, and from 400 °C to 800 °C, the fractal dimension increases with the increase in temperatures due to the higher the temperature, the higher the damage degree of the material, the more serious the deterioration of mechanical properties. In addition, Figure 10 also shows that the fractal dimension is the largest under the condition of 0.2% fiber content, followed by the condition of 0.4% fiber content, and the fractal dimension is the smallest under the condition of 0.1% and 0.3% fiber content.

In conclusion, the fractal dimension of BFRC fragments are affected by the coupling effects of temperature, impact velocity and fiber content. Due to the influence of elevated temperature degradation and impact energy, the temperature and the impact velocity have a significant correlation with the fragmentation characteristics of the fragments, and the higher the temperature, the higher the impact velocity, and the more seriously the specimen failure. BFRC has more stable performance under elevated temperature and impact load coupling due to high temperature resistance and toughening effect of basalt fibers. The mechanism of basalt fiber reinforcement is that when temperature and impact velocity are low, only microscopic cracks that consume less energy have a practical effect on breaking concrete, and continuous expansion and penetration of these microcracks causes the failure of concrete before the absorption of energy increases sufficiently to cause other new cracks and become a major crack; when temperature and impact velocity are high, the absorption of energy by concrete reaches a high level before the deep expansion and penetration of cracks, which allows more cracks to expand and participate in the crushing process, resulting in a smaller fracture scale and a larger fractal dimension. The addition of basalt fibers could prevent the expansion of microcracks, so that the energy absorbed by concrete is used to generate new microcracks to participate in the crushing process. Therefore, the crushing scale of BFRC is smaller and the fractal dimension is larger under the elevated temperature and high impact velocity.

## 4. Energy Dissipation Analysis

According to the thermodynamic theorem, the energy transformation is the essential characteristics of physical change processes of the matter, while the material destruction is the instability phenomenon driven by the energy [21]. The process of concrete destruction is actually an irreversible process of energy consumption when exchanging energy with the outside. The energy dissipation of BFRC after exposure to elevated temperatures under impact loading has changed significantly compared with that at room temperature, but there is little research in this field. In this paper, the energy dissipation of BFRC after exposure to elevated temperatures under impact loading on the basis of the principle of SHPB energy dissipation, and the influence of elevated temperature, impact velocity and fiber content on dissipated energy of BFRC were analyzed.

### 4.1. Energy Dissipation Principles of SHPB Test

According to the one-dimensional stress wave theory and the energy conservation law, the calculation equation of incident energy, reflected energy and transmitted energy of SHPB test is

(4) $$\left\{ \begin{array}{l} W_{I}=\frac{A_{0}C_{0}}{E_{0}}\int \sigma_{I}^{2}{(t)\mathrm{dt}=A}_{0}C_{0}E_{0}\int \varepsilon_{I}^{2}(t)\mathrm{dt}\\ W_{R}=\frac{A_{0}C_{0}}{E_{0}}\int \sigma_{R}^{2}{(t)\mathrm{dt}=A}_{0}C_{0}E_{0}\int \varepsilon_{R}^{2}(t)\mathrm{dt}\\ W_{T}=\frac{A_{0}C_{0}}{E_{0}}\int \sigma_{T}^{2}{(t)\mathrm{dt}=A}_{0}C_{0}E_{0}\int \varepsilon_{T}^{2}(t)\mathrm{dt} \end{array}\right.$$

where *W_I_*, *W_R_* and *W_T_* are the incident energy, reflected energy and transmitted energy, respectively. $\sigma_{I}$, $\sigma_{R}$ and $\sigma_{T}$ are the stress-time history of the incident wave, reflected wave and transmitted wave respectively. $\varepsilon_{I}$, $\varepsilon_{R}$ and $\varepsilon_{T}$ are the strain–time history of the incident wave, reflected wave and transmitted wave, respectively. $A_{0}$, $C_{0}$ and $E_{0}$ are the cross-sectional area, wave velocity of the elastic bar and the elastic modulus, and the value of $C_{0}$ is 5181 m/s.

Omitting the energy loss caused by the friction between the specimen and section of incident/transmitted bar, the total energy absorbed by the concrete specimen during the impact crushing process is

(5) $${W=W}_{I}-(W_{R}{+W}_{T}){=A}_{0}C_{0}E_{0}\int {(\varepsilon }_{I}^{2}{(t)-\varepsilon }_{R}^{2}{(t)-\varepsilon }_{T}^{2}(t))\mathrm{dt}$$

where *W* is the total energy absorbed by the concrete specimen during the impact crushing process, which is mainly divided into four parts [22]: fracture energy *W_F_*, mainly used for the formation of the fracture surface; damage energy *W_D_*, mainly used for the crack expansion, the microcrack propagation and so on; kinetic energy *W_K_* of broken blocks; other forms of dissipated energy *W_O_*, such as thermal energy, radiant energy, etc. Among them, both ***W_D_*** and ***W_O_*** are very small, which can be omitted. Then, the total absorption energy *W* can be written as

*W* = *W_F_ + W_K_* (6)

According to the research result of Zhang [23], the relationship between the *W_K_* and *W* in the SHPB test can be expressed as

*W_K_/W* = (0.69*v* + 0.22)/100 (7)

where *v* is the impact velocity, m/s. The impact velocities of this test were 5.4 m/s, 8.8 m/s, and 11.3 m/s; substitute the velocities to the Equation (7), and the result shows that the *W_K_* is 3.95%, 6.29%, 8.02% of *W,* respectively, and all are less than 10%, so we can draw a conclusion that the proportion of kinetic energy is small, and that most of the energy is used for the fracture failure of specimens. 

In order to eliminate the influence of the specimen size, the energy dissipation density, namely the absorbed energy per unit volume of the specimen, was introduced to reflect the energy consumption characteristics of concrete materials

(8) ζ=W/V

where ζ and *V* are the energy dissipation density and the volume of concrete specimens, respectively.

### 4.2. Calculation and Analysis of Energy Dissipation

The incident energy *W_I_*, absorbed energy *W* and energy consumption density ζ of BFRC calculated by Equations (4)–(8) under different working conditions are shown in Table 3. The relation curve of absorbed energy with temperatures and impact velocities is shown in Figure 10a (the fiber content is 0.2%). The relation curve of absorbed energy with temperatures and fiber contents is shown in Figure 10b (the impact velocity is 8.8 m/s).

It can be seen from Table 3 and Figure 10a that the total absorbed energy of BFRC increases with the increase in impact velocity for the same fiber content. This can be explained as follows: the energy expended in the creation of new cracks is much higher than the energy needed to crack extension, the larger the impact velocity, the more the number of microcracks, the more absorbed energy, the more serious the fracture degree of the specimen, and the smaller the size of the fragments.

As shown in Table 3 and Figure 10b, when the impact velocity remains unchanged, the absorbed energy increases first and decreases later with the increase in fiber content; the absorbed energy reaches the maximum when the fiber content is 0.2%. This phenomenon can be explained as follows: when the fiber content is less than 0.2%, basalt fibers are randomly distributed in the concrete and form a network, so it can prevent the appearance and development of microcracks, improve the toughness of concrete, but when the fiber content exceeds 0.2%, more basalt fibers tend to agglomerate in the concrete mixing process, and then form pores in the concrete, stress concentration tends to occur around the pores under the action of external forces, resulting in the reduction in concrete strength and the ability to absorb energy. When the fiber content remains unchanged, the absorbed energy decreases with the rise in temperature. The main reasons for this phenomenon are as follows: the free and bound water inside concrete evaporate, and the hydrates dehydrate and decompose after exposure to elevated temperature, leading to the continuous development of cracks, the strength of the hydrates reduces, and the performance of the concrete deteriorates; consequently, concrete strength and the energy-dissipating capacity reduce. For 11.3 m/s impact velocity, there is no significant decrease in absorbed energy when the temperature changes from 20 °C to 200 °C, mainly because of minor temperature damage in the concrete after exposure to 200℃, when the impact velocity is high, the effect of temperature damage is less obvious [24,25].

### 4.3. The Relationship Between Energy Intensity and Fractal Dimension

The energy dissipation density is the energy consumed by the unit of volume of concrete, and it reflects the capacity of energy dissipation or crushing resistance of concrete. Under the action of external impact load, energy dissipation is the essential internal reason that leads to concrete failure, while the fracture form is the main external reason [16]. Therefore, there is an essential relation between the energy dissipation density and the fractal dimension of concrete fragments in the process of impact crushing.

**Figure 11:**
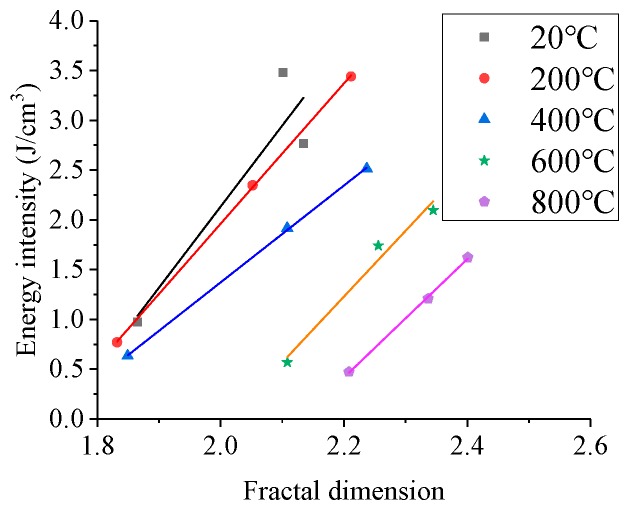
The relationship between ζ and *D*(the fiber content is 0.2%).

The relation curve between the energy dissipation density ζ and fractal dimension *D* of BFRC specimens under the 0.2% fiber content is shown in Figure 11, which is fitted according to the linear equation. The expressions and correlation coefficients R are shown in Table 4.

As shown in Figure 11 and Table 4, under the same temperature, the energy dissipation density of BFRC increases linearly with the increase in fractal dimension, and linear formula is fitted, the math expressions is ζ=aD+b, where a and b are fitting coefficients. The squares of the correlation coefficient are 0.8541, 0.9998, 0.9997, 0.9750, 0.9991, indicating that there is a good linear relationship between the energy dissipation density and fractal dimension. When the temperature rises from 20 °C to 200 °C, the energy dissipation density and fractal dimension change little, but when the temperature rises from 400 °C to 800 °C, the energy dissipation density decreases, and the fractal dimension increases significantly. The reason for this phenomenon is that only minor temperature damage occurs in concrete after exposure to 200 °C, but when the temperature reaches 400 °C–800 °C, the temperature damage becomes obvious and increases with the increasing temperatures. It leads to the deterioration of concrete performance, the decrease in energy absorption capacity, and the increase in crushing degree.

**Figure 12:**
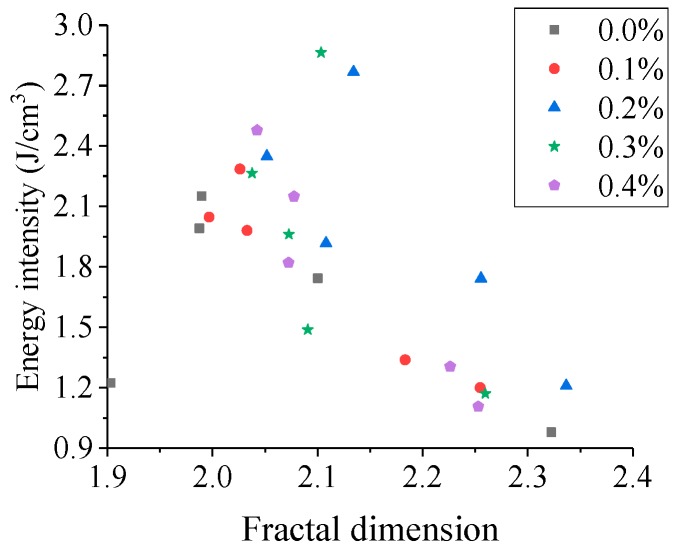
The relationship between ζ and *D* (the impact velocity is 8.8 m/s)**.**

The relation curve between energy dissipation density **ζ** and fractal dimension *D* under a certain impact velocity is shown in Figure 12.

From Figure 12, it can be observed that under the same impact velocity, the energy dissipation density and the fractal dimension increases first and decreases later with the increasing fiber content, and the maximum values occur when the fiber content is 0.2%, but there is no obvious linear relationship between energy dissipation density and fractal dimension. This phenomenon can be explained as follows: when the impact velocity and fiber content remain unchanged, the energy dissipation density decreases with the increase in temperature, but the fractal dimension does not change significantly at 20 °C–400 °C, and increases obviously at 600 °C–800 °C; the changes at different temperatures are not synchronized, so the linear relationship between them no longer exists.

## 5. Conclusions

In this research, we investigated the fractal characteristics and energy dissipation of BFRC through SHPB tests. The effect of fiber contents, temperatures and impact velocities were studied. Based on the experimental results, we obtain the following discussions and conclusions:

(1) Temperature and impact velocity have a significant influence on the scale-mass distribution of the fragments. The higher the heating temperature and the greater the impact velocity, the more serious the specimen fragmentation, and the larger the mass proportion of small and medium-sized fragments.

(2) The process of BFRC destruction from the microscopic damage to the macroscopic crushing is characterized by fractal properties. The fractal dimension increases significantly with the increase in impact velocity, and generally increases with the increase in temperature. When the temperature increases from 20 °C to 200 °C, the fractal dimension does not change obviously, when the temperature increases from 400 °C to 800 °C, it increases significantly.

(3) The absorbed energy increases with the increasing impact velocity and decreases with the increasing temperature, and increases first and decreases later with fiber content; the maximum values occur when the fiber content is 0.2%. When the impact velocity is high, there is no significant decrease in absorbed energy when the temperature changes from 20 °C to 200 °C.

(4) The process of concrete impact crushing is a fractal evolution process driven by external energy, so there is an essential relation between the energy dissipation density and fractal dimension of concrete fragments. There is a good linear relationship between energy dissipation density and fractal dimension under the same temperature and fiber content. When the impact velocity remains unchanged, the energy consumption density and fractal dimension are not synchronized with the increase in fiber content, so there is no obvious linear relationship between them.

## Figures and Tables

**Table 1:** Mixing Proportions of Concrete Specimens.

Item	Water	Cement	Fine Aggregate	Coarse Aggregate	Fly Ash	Water Reducer
Proportion	0.56	1	2.03	3.80	0.40	0.02

**Table 2:** Basalt fibers performance parameters.

Diameter (μm)	Length (mm)	Tensile Strength (MPa)	Elasticity Modulus (GPa)	Fracture Elongation (%)	Density (kg/m^3^)
17.4	12	≥2000	≥85	2.5	2699

**Table 3:** Calculation results of *W_I_, W* and ζ under different conditions.

Fiber Content (%)	*T* (°C)	v (°C)	*W_I_* (J)	*W* (J)	*W_F_* (J)	*W_K_* (J)	*W_F_/W* (%)	*W_K_/W* (%)	ζ (J/cm^3^)
0	20	8.8	310.60	95.37	89.37	6.00	93.71	6.29	2.15
0	200	8.8	315.84	88.35	82.79	5.56	93.71	6.29	1.99
0	400	8.8	317.64	78.05	73.14	4.91	93.71	6.29	1.74
0	600	8.8	293.61	55.65	52.14	3.50	93.71	6.29	1.22
0	800	8.8	311.34	45.00	42.16	2.83	93.71	6.29	0.98
0.1	20	8.8	301.67	104.80	98.21	6.59	93.71	6.29	2.28
0.1	200	8.8	330.82	93.31	87.44	5.87	93.71	6.29	2.05
0.1	400	8.8	341.95	89.95	84.29	5.66	93.71	6.29	1.98
0.1	600	8.8	285.91	62.01	58.10	3.90	93.71	6.29	1.34
0.1	800	8.8	360.78	56.39	52.84	3.55	93.71	6.29	1.20
0.2	20	5.4	95.92	45.10	43.32	1.78	96.05	3.95	0.97
0.2	20	8.8	323.40	128.49	120.41	8.08	93.71	6.29	2.77
0.2	20	11.3	487.22	164.62	151.43	13.20	91.98	8.02	3.48
0.2	200	5.4	86.84	36.65	35.21	1.45	96.05	3.95	0.77
0.2	200	8.8	286.95	110.29	103.35	6.94	93.71	6.29	2.35
0.2	200	11.3	509.43	165.85	152.55	13.30	91.98	8.02	3.44
0.2	400	5.4	80.17	29.67	28.50	1.17	96.05	3.95	0.63
0.2	400	8.8	309.18	91.74	85.96	5.77	93.71	6.29	1.92
0.2	400	11.3	433.17	118.80	109.28	9.52	91.98	8.02	2.51
0.2	600	5.4	92.46	26.98	25.91	1.06	96.05	3.95	0.57
0.2	600	8.8	330.52	84.46	79.15	5.31	93.71	6.29	1.74
0.2	600	11.3	500.00	99.70	91.71	7.99	91.98	8.02	2.10
0.2	800	5.4	106.69	23.11	22.20	0.91	96.05	3.95	0.47
0.2	800	8.8	280.08	58.27	54.60	3.67	93.71	6.29	1.21
0.2	800	11.3	525.50	77.95	71.70	6.25	91.98	8.02	1.62
0.3	20	8.8	357.75	127.30	119.29	8.01	93.71	6.29	2.86
0.3	200	8.8	276.77	100.25	93.94	6.31	93.71	6.29	2.26
0.3	400	8.8	302.55	88.89	83.29	5.59	93.71	6.29	1.96
0.3	600	8.8	273.07	65.43	61.32	4.12	93.71	6.29	1.49
0.3	800	8.8	285.30	53.27	59.29	3.98	93.71	6.29	1.17
0.4	20	8.8	333.95	110.65	103.69	6.96	93.71	6.29	2.48
0.4	200	8.8	289.74	94.62	88.66	5.95	93.71	6.29	2.15
0.4	400	8.8	325.44	83.36	78.11	5.24	93.71	6.29	1.82
0.4	600	8.8	294.74	59.31	64.95	4.36	93.71	6.29	1.30
0.4	800	8.8	363.00	50.51	47.33	3.18	93.71	6.29	1.11

**Table 4:** The fitting formula expressions between ζ and *D* under different temperatures.

Temperature (°C)	Fitting Equation Expressions	*R*	*R* ^2^
20	ζ=8.1346D−14.135	0.9242	0.8541
200	ζ=7.0423D−12.122	0.9999	0.9998
400	ζ=4.8634D−8.354	0.9998	0.9997
600	ζ=6.6043D−13.299	0.9874	0.9750
800	ζ=5.9401D−12.651	0.9995	0.9991
